# Diagnosing brain tumours by routine blood tests using machine learning

**DOI:** 10.1038/s41598-019-51147-3

**Published:** 2019-10-09

**Authors:** Simon Podnar, Matjaž Kukar, Gregor Gunčar, Mateja Notar, Nina Gošnjak, Marko Notar

**Affiliations:** 1 Division of Neurology, University Medical Centre Ljubljana, Ljubljana, Slovenia; 2 Faculty of Computer and Information Science, University of Ljubljana, Ljubljana, Slovenia; 3 Smart Blood Analytics Swiss SA, Chur, Switzerland

**Keywords:** Diagnostic markers, CNS cancer

## Abstract

Routine blood test results are assumed to contain much more information than is usually recognised even by the most experienced clinicians. Using routine blood tests from 15,176 neurological patients we built a machine learning predictive model for the diagnosis of brain tumours. We validated the model by retrospective analysis of 68 consecutive brain tumour and 215 control patients presenting to the neurological emergency service. Only patients with head imaging and routine blood test data were included in the validation sample. The sensitivity and specificity of the adapted tumour model in the validation group were 96% and 74%, respectively. Our data demonstrate the feasibility of brain tumour diagnosis from routine blood tests using machine learning. The reported diagnostic accuracy is comparable and possibly complementary to that of imaging studies. The presented machine learning approach opens a completely new avenue in the diagnosis of these grave neurological diseases and demonstrates the utility of valuable information obtained from routine blood tests.

## Introduction

Diagnosis of brain tumours is usually based on the patient’s history, neurological examination and neuroimaging findings. The obstacles in diagnosis are often nonspecific clinical presentations^1^ and the relative rarity of brain tumours (estimated annual incidence rate in the USA: 29/100,000 population)^2^. Therefore, in order not to miss a diagnosis, physicians need to order a large number of neuroimaging studies, many of which turn out to be negative. Widespread referrals for neuroimaging are costly, not just economically^3^ but also medically due to false positive studies^4^. As the majority of brain tumours are initially diagnosed in emergency departments, using predominantly computed tomography (CT) imaging, ionization radiation exposure of patients is also a concern^5^. Diagnostic accuracy of neuroimaging for brain tumours is also not ideal^6,7^. As more effective treatments for brain tumours are developed, their early diagnosis may become even more important^8^. Although neuroimaging studies will unquestionably remain pivotal in the future, there is a definite need for cheap and efficient alternative diagnostic approaches that are useful in patients with headache and other common neurological presentations of brain tumours^1^.

Tumours secrete various substances^9^, and the body reacts to tumour growth^10^, both of which affect body fluid composition. Physicians are able to extract only a small fraction of information hidden in routine blood test results^11^. Therefore, it is valuable to analyse routine blood test results more closely to try to find evidence of neoplastic growth. Because machine learning (ML) can recognise subtle patterns in data, it is ideal for this task. We previously demonstrated that the ML model largely outperformed experienced clinicians in diagnosing haematological disorders^11^. Brain tumours also seem suitable for the application of ML because neuroimaging presents a solid reference test for their diagnosis, which is essential for building good predictive models.

The present study aimed to determine the diagnostic accuracy of the ML model for diagnosing brain tumours from the results of routine blood tests. After building a model using a large database of neurological patients, we assessed the sensitivity of this approach in a group of patients with newly diagnosed brain tumours and the specificity in a cohort of consecutive control patients evaluated at the neurological emergency service by routine blood tests and neuroimaging studies. We also determined blood parameters that were most important in the diagnosis of brain tumours by the ML model.

## Materials and Methods

### Patients and controls

**Figure 1:**
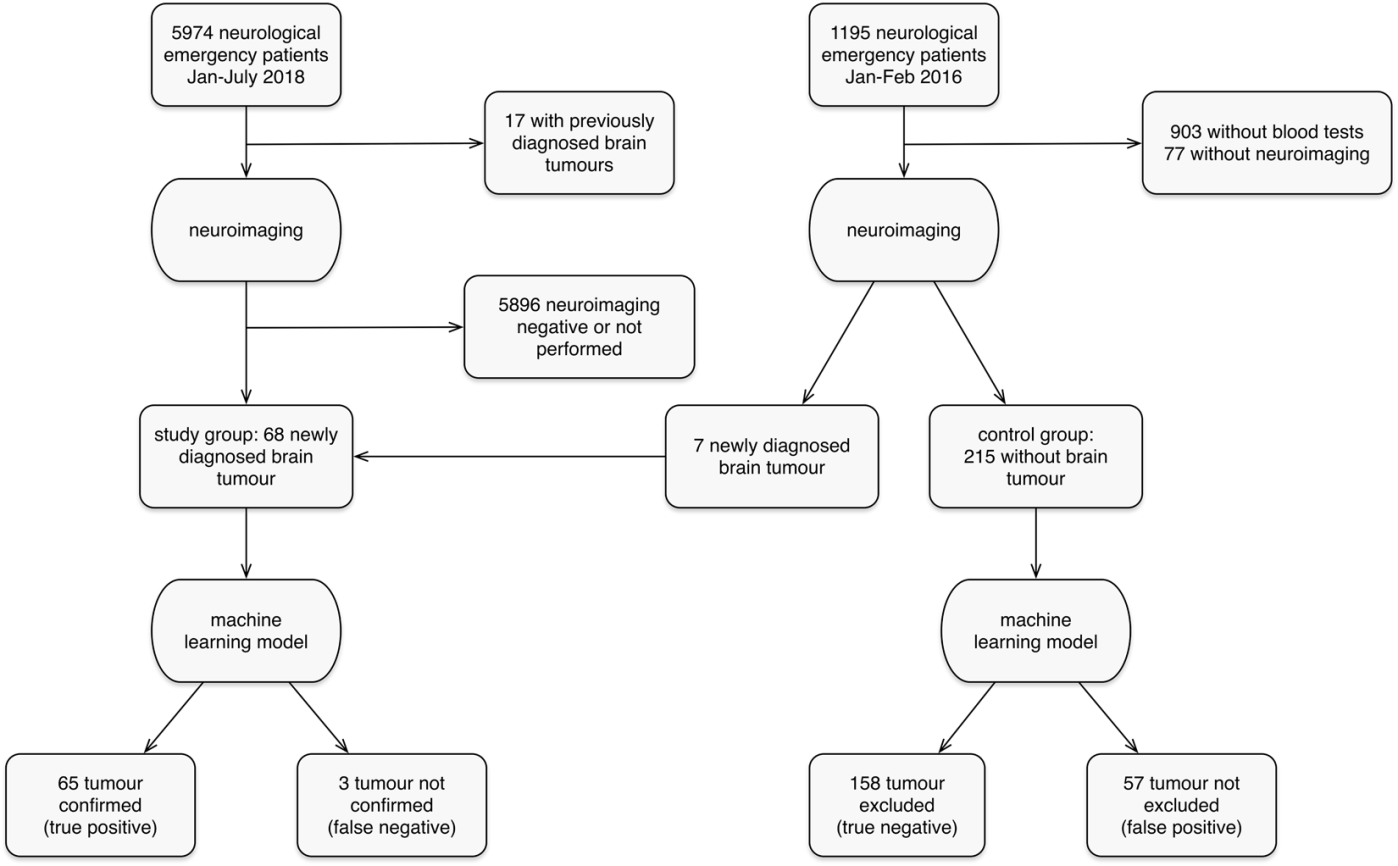
Flow chart of patients included in the validation process.

We obtained data from several groups of patients. (1) The “training group” included retrospective patient data obtained from March 2012 to December 2017 at the Division of Neurology, University Medical Centre Ljubljana (UMCL), Slovenia. (2) The “validation study group” included retrospective data of consecutive patients with newly diagnosed intrathecal tumours recruited at the same institution from January to July 2018. Patients with previously diagnosed tumours, with an uncertain tumour diagnosis, and patients treated with antipsychotic, antiepileptic, antibiotic or corticosteroid medications were excluded. (3) The “validation control group” included consecutive patients who were evaluated at the neurological emergency service of the same institution from January to mid-February 2016. These data were available from a previous analysis and were excluded from the training group. Patients with newly diagnosed brain tumours from this group were added to the validation study group (Fig. 1).

In all groups, we collected data on age, sex, blood test results and the ICD10-encoded final diagnoses. Only patients with neuroimaging studies were included in the validation group (both study and control patients). Additionally, symptom duration, tumour dimensions, and neuroimaging diagnoses were also collected for the validation study group. From dimensions measured on MR, we calculated tumour volumes. We analysed all blood parameters available in the electronic medical records using our ML algorithm.

### The smart blood analytics (SBA) machine learning (ML) algorithm

The SBA algorithm is an ML pipeline consisting of several processing stages, corresponding to phases 2–6 of the CRISP-DM standard^12^: (1) data acquisition: acquiring raw data from the database; (2) data filtering: using only blood test results obtained before treatment and the final diagnosis as the training subset; (3) data preprocessing: canonizing blood parameters (matching them with our reference blood parameter database, filtering out outliers, recalculation to SI units); (4) data modeling: building the predictive model using ML algorithms; (5) evaluation: evaluating the model with stratified 10-fold cross-validation; (6) deployment of the successfully evaluated model through the SBA website^13^. As the principal ML algorithm, we used the extreme gradient boosting machine (XGBoost)^14–16^, which is currently one of the most popular tools^17^.

### Evaluation of predictive models

The models were automatically evaluated using stratified ten-fold cross-validation. The results were used in a statistical comparison of the model’s performance on all training cases, aggregated into confusion matrices and to calculate performance measures (sensitivity, specificity, receiver operating characteristic (ROC) curve). To obtain “binary” performance measures, we formed the “tumour” and the “no tumour” groups. To better understand the model’s behaviour within the tumour group, we reviewed its confusion matrix by confronting patients’ actual diagnoses (determined with neuroimaging) with their predicted diagnoses (predicted from blood tests).

### Blood parameter frequency and importance

We analysed the relative frequencies of blood parameters and explored the effect of missing (not measured) parameters on predictive accuracy. The predictive model performance was evaluated by incrementally including blood parameters, starting with the most frequent ones. The resulting learning curve depicted the relation between the number of parameters and predictive accuracy. XGBoost predictive models explicitly calculate blood parameter importance scores^18^ that can be used for ranking parameters. We determined the 40 most important blood parameters measured in both the training and validation datasets. For each blood parameter, we also calculated the relative frequency, reference range, and median values for all groups. All blood parameter values (reference ranges, medians) were centred and scaled according to reference ranges. We compared blood parameter distributions in groups by the nonparametric k-sample Anderson-Darling (AD) test^19^. If the null hypothesis was rejected (α = 0.05), we also calculated the “common language” effect size^20^.

### Statistics

According to STARD criteria^21^, we calculated the ML model sensitivity, specificity and accuracy in the validation group, with neuroimaging as a reference standard. In addition, for sensitivity and specificity, the 95% Agresti-Coull binominal confidence intervals were calculated^22^. All tests were performed at a significance level of α = 0.05 (two-sided).

## Results

**Figure 2:**
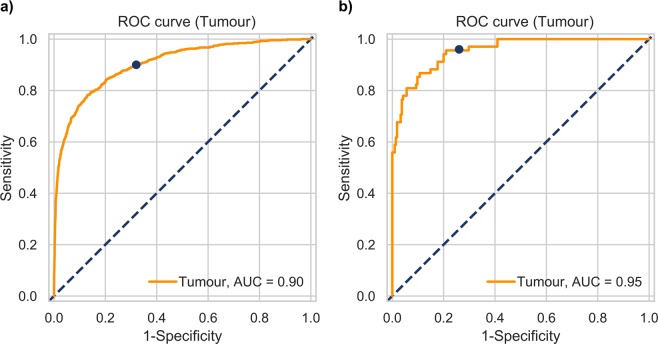
ROC curves for tumour diagnosis calculated from (**a**) the training data using ten-fold stratified cross-validation and from (**b**) the validation data. The blue dot depicts the point corresponding to the selected threshold value.

The training sample included 15,176 neurological patients, 701 of whom had intrathecal tumours, most often malignant (Table 1). Table 2 shows the sensitivity, specificity and accuracy (@k = 1, 3, 5, 10) of the original model, predicting all 87 diagnoses. Figure 2a shows the ROC curve of the ML model adapted for predicting only the presence/absence of tumours, obtained with 10-fold stratified cross-validation in training data. The selected threshold value of 0.025 in the training dataset resulted in a sensitivity of 90% (95% confidence interval (CI), 88–92%), a specificity of 68% (95% CI, 67–69%) and an accuracy of 69% (95% CI, 68–70%).

**Table 1:** Characteristics of data used for the training and validation of the machine learning model.

	Training group	Validation group	
		Tumour	Control
Recruitment period	2012/03–2017/12	2018/01–2018/07	2016/01–2016/02
Inclusion criteria	Blood tests & diagnoses	Blood tests & neuroimaging	Blood tests & neuroimaging
Number of patients	15, 176	68	215
Number of men (%)	7285 (48%)	34 (50%)	115 (53%)
Age range (median) years	18–103 (67)	19–86 (66)	24–97 (74)
The most frequent ICD diagnosis (I63 – cerebral infarction)	34%	0%	49%
Number of tumour patients	701	68	0
Number of blood parameters	295	110	138
Missing parameter values	83%	81%	84%
Tumour diagnoses			
Malignant neoplasm of brain - C71	496 (70.8%)	56 (82.4%)	0 (0%)
Benign neoplasm of the brain and other parts of the CNS - D33	26 (3.7%)	0 (0%)	0 (0%)
Neoplasm of uncertain behaviour of the brain and CNS - D43	51 (7.3%)	0 (0%)	0 (0%)
Malignant neoplasm of the meninges - C70	36 (5.1%)	0 (0%)	0 (0%)
Benign neoplasm of the meninges D32	63 (9.0%)	9 (13.2%)	0 (0%)
Neoplasm of uncertain behaviour of the meninges - D42	5 (0.7%)	0 (0%)	0 (0%)
Malignant neoplasm of the spinal cord, cranial nerves and other parts of the CNS - C72	24 (3.4%)	3 (4.4%)	0 (0%)

**Table 2:** Basic performance indicators with Agresti-Coull binomial confidence intervals (CIs) as obtained from the training dataset (n = 15,176 neurological patients) with ten-fold cross-validation and in the validation dataset (68 brain tumour patients and 215 control patients).

k	Sensitivity@k ± binominal CI		Specificity@k ± binominal CI	
	Training dataset	Validation dataset	Training dataset	Validation dataset
1	0.51 ± 0.037	0.51 ± 0.116	0.98 ± 0.002	0.99 ± 0.018
3	0.66 ± 0.035	0.69 ± 0.108	0.93 ± 0.004	0.93 ± 0.035
5	0.78 ± 0.031	0.79 ± 0.097	0.83 ± 0.006	0.82 ± 0.052
10	0.95 ± 0.016	0.99 ± 0.043	0.38 ± 0.008	0.23 ± 0.056

@k – prediction is correct if the actual tumour diagnosis is within the first k predicted diagnoses.

Of the 5974 patients who presented to UMCL during the tumour patient recruitment period of 7 months, 85 patients were identified. After exclusion of 17 patients with known tumour diagnoses, 68 patients with newly diagnosed intrathecal tumours were further analysed (Fig. 1). They were symptomatic for a few hours to six months (median, ten days), and their tumour volumes varied from 2 to 145 cm^3^ (median, 19 cm^3^). Tumour MR diagnoses were glioma in 22 patients, glioblastoma in 15, metastases in 14, meningioma in 11, schwannoma in 2, and ependymoma, neuroblastoma, pituitary adenoma, and craniopharyngioma in 1 patient each. The majority of patients were diagnosed with malignant brain tumours (Table 1).

During the recruitment period of one and a half months, 1195 patients were evaluated at the UMCL neurological emergency service. Routine blood tests were performed in 292 patients, however no neuroimaging was performed in an additional 77 patients, resulting in 215 control patients (Fig. 1). Their final diagnoses were as follows: ischemic stroke in 105, epileptic seizures in 20, subarachnoid haemorrhage in 19, intracerebral haemorrhage in 14, transient ischemic attack (TIA) in 10, headache in 7, dementia in 3, etc. During recruitment of this group, 7 patients with brain tumours (3 meningiomas) were also diagnosed and were added to the validation study group.

Sensitivity, specificity and accuracy (@k = 1, 3, 5, 10) in the validation group are shown in Table 2 (see Supplementary Table S1 for TP, FN, TN and FP values). Using the threshold for the ROC curve selected in the training data, the sensitivity of the model was 96% (95% CI, 91–100%), the specificity was 74% (95% CI, 68–79%) and the accuracy was 79% (95% CI, 74–84%) (Fig. 2b). One-third of meningiomas (7 out of 21) were predicted to be malignant brain tumours (C71).

### Evaluating blood parameter frequency and importance in the training group

Using 50 of the most frequent blood parameters measured in more than half of the patients, the diagnostic accuracy was 50%. Maximum accuracy of 54% was reached with 100 blood parameters; the contribution of the remaining 195 less frequently measured blood parameters was negligible.

**Figure 3:**
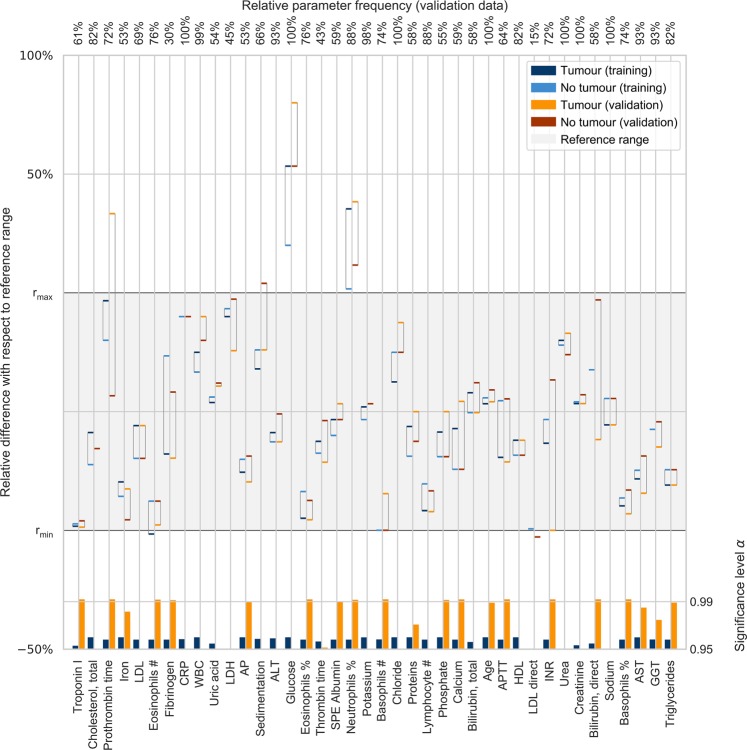
Group median values of the 40 most important routine blood parameters, centred and scaled to reference intervals. Groups (tumour/no tumour) within the datasets (training/validation) were evaluated by the Anderson-Darling test. The significance levels (0.95 and 0.99) of the test results are depicted at the bottom.

Blood parameters with the highest discriminative power are shown in Fig. 3. Only the first 29 parameters were relatively important. The majority of parameters in the validation group fell within the reference range (Fig. 3).

When comparing training and validation data, for 12 of 295 blood parameters we can reject the null hypothesis that they were sampled from the same distribution, but even for them, the “common language” effect size^20^ was negligible.

## Discussion

Our results confirmed that brain tumour diagnosis is indeed feasible from routine blood tests by the application of ML to data obtained from neurological patients. This approach challenges standard procedures and opens a completely new avenue to diagnose these devastating neurological diseases. We adapted the predictive model to specifically aim at brain tumour diagnosis, constructed ROC curves, and determined the threshold value. In our training data, the cross-validated AUC (area under the curve) was 0.90 (Fig. 2a), and in our validation data AUC reached 0.95 (Fig. 2b). AUC values above 0.90 are considered excellent^23^. In order not to miss patients with tumours, it is desirable to have a high sensitivity, coupled with reasonable specificity. In the validation group, the sensitivity of the adapted tumour model was 96%, and the specificity was 74% at the previously selected threshold. Sensitivity (90%) and specificity (68%) were slightly lower for cross-validation in the training group. This difference is likely due to better data quality in the validation group. We excluded patients with previously diagnosed brain tumours (17/87 = 22%) from our validation group but could not do the same from the training sample. The majority of previously diagnosed patients were treated by surgery, irradiation and chemotherapy. In addition, they usually use other medications for symptom management. These interventions change blood biochemistry and may degrade the diagnostic performance of the ML model. Similar problems can occur in patients treated with antiepileptic, antipsychotic, antibiotic, or corticosteroid medications. Our model is therefore useful for the primary diagnosis of brain tumours in patients who are not taking medications that could have major effects on blood composition.

We believe that our results present a paradigm shift in the clinical diagnosis of brain tumours. Patients presenting with headache or other nonspecific complaints, possibly caused by a brain tumour, may now first have routine blood tests assessed by the developed ML model. All patients with predicted brain tumours would then have neuroimaging studies to confirm the diagnosis and obtain information on the location, size and characteristics of brain tumours, which are necessary for further management.

The diagnostic performance of imaging studies is also not perfect in brain tumours. In a recent study including 127 patients, the sensitivity for the diagnosis of brain tumours was 88% for CT, 89% for magnetic resonance imaging (MRI), and 93% for positron emission tomography (PET)^24^. In another recent study, the sensitivity of MRI for the detection of brain metastases with diameters >2 mm evaluated by four radiologists (two residents) varied from 85% to 95% (average, 89%)^6^. The diagnostic performance of our predictive model is therefore similar to that of imaging studies.

We obtained blood samples from patients immediately after they presented to our neurological service with a median symptom duration of 10 days. The tumour volume on presentation varied, with the smallest of only 2 cm^3^. This observation suggests that our algorithm is also useful in the early symptomatic phase when tumours are small and easier to miss on imaging studies. We do not have data on the ability of our model to diagnose presymptomatic brain tumours. However, we previously reported a patient in whom our haematological model diagnosed plasmacytoma from blood parameters obtained two years before the first symptoms^11^. Nevertheless, due to the low prevalence of brain tumours in the general population, our model is probably not useful for presymptomatic screening.

Our model was also not useful for differentiation among primary malignant brain tumours, brain metastases and meningiomas. This differential ability, however, is not particularly relevant because all patients with suspected space-occupying lesions need neuroimaging studies. In patients with suspected brain metastasis, our other ML models could be employed (e.g., pulmonary, gastrointestinal, etc.) to noninvasively identify the primary tumours. Validation data also included 3 other intrathecal nerve tumours: 2 acoustic schwannomas and 1 ependymoma of the cauda equina. Although the diagnostic accuracy was low, the group was too small for conclusions (Table 1).

Another potential use of our algorithm is differentiation of brain tumours from tumour mimics, e.g., demyelinating disease, primary vasculitis, tuberculoma, vascular lesions, neurosarcoidosis, etc., which often pose a diagnostic challenge for neuroimaging studies^25^. Similarly, differentiation of tumours from radiation necrosis can also be difficult. Further appropriately designed studies are needed to validate ML predictive models in these situations.

Some routine blood parameters proved to be more important in our model than other parameters were. However, looking at the 40 most important parameters (e.g., troponin I, total cholesterol, prothrombin time, iron, etc.) does not provide physicians with clues regarding brain tumour diagnosis (Fig. 3). Troponin is not related to tumours, but it is likely important to exclude patients with stroke^26^. Importantly, in our model, these parameters are mainly within the normal range, but their values differ considerably between the tumour group and other neurological conditions (e.g., stroke, epilepsy, etc.). Glucose and neutrophils are the only two that fall outside the normal range, and both are significantly more elevated in the tumour group compared to the nontumour group (Fig. 3). Interestingly, lymphocytes, eosinophils and basophils were all decreased in the tumour group, indicating a possible role for tumours in the down-regulation of the adaptive immune cells that fight against tumours.

Our study has some limitations. First, we performed this analysis on data obtained in a single centre, which might limit its generalizability. However, using standardised and approved procedures, reagents and technology, we would expect similar laboratory blood test results elsewhere. Second, we included in the analysis of the validation group only a minor proportion of neurological patients because, in the opinion of neurologists, the majority did not require blood testing or neuroimaging studies. Third, the study was retrospective, which limited the scope of information obtained from our patients. However, for our purpose, we mainly needed results of routine blood tests and accurate neuroradiological diagnoses that were available for all patients with brain tumours. Finally, we did not obtain the final histological diagnoses of brain tumours. In our opinion, this was not essential because the model is only aimed at deciding whether to proceed with imaging, and for this purpose, neuroimaging study findings were sufficient.

The study also has several strengths. First, we analysed data from a large number of patients (>15,000) with good data quality for blood tests and diagnoses. Second, neurologists diagnosed all patients, and neuroradiologists read and interpreted all neuroimaging studies, which assured high quality of the diagnoses. Third, we used state-of-the-art ML algorithms that are able to develop the best predictive models. Finally, we validated our model in both the whole training group (15,176 patients) and the validation group (283 patients) and obtained consistent results.

The present study demonstrated that brain tumours can be efficiently diagnosed from the results of routine blood tests. The SBA brain tumour ML model was able to extract subtle prognostic data from blood test results that are hidden even from the most experienced clinicians. We believe that our results present a real paradigm shift in the approach that physicians can use to diagnose brain tumours.

## Supplementary information

Supplementary Table S1

## Data Availability

The predictive model is available at www.smartbloodanalytics.com upon registration and reasonable request for its use. The study was approved by the Slovenian National Medical Ethics Committee No. 0120-718/2015/5.
