# Supplementary material for: Diagnosing brain tumours by routine blood tests using machine learning
Source: Sci Rep. 2019 Oct 9;9:14481. doi: 10.1038/s41598-019-51147-3 (PMC6785553; doi:10.1038/s41598-019-51147-3)
Supplement: Supplementary file 1 — Supplementary Table S1 [file 41598_2019_51147_MOESM1_ESM.pdf]

Diagnosing brain tumours by routine blood tests using machine learning

Simon Podnar,*[1] Matjaž Kukar,[2,4] Gregor Gunčar,[3,4] Mateja Notar,[4] Nina Gošnjak,[1] Marko Notar[4]

[1]Division of Neurology, University Medical Centre Ljubljana, Slovenia

[2]Faculty of Computer and Information Science, University of Ljubljana, Slovenia

[3]Faculty of Chemistry and Chemical Technology, University of Ljubljana, Slovenia

[4]Smart Blood Analytics Swiss SA, Chur, Switzerland.

Supplementary Information

Supplementary Table S1. TP (true positive), FN (false negative), TN (true negative) and FP (false positive) for model performance on training and validation dataset. @k – prediction is correct if the actual tumour diagnosis is within the first k predicted diagnoses.

	training dataset				validation dataset			
k	TP	FN	TN	FP	TP	FN	TN	FP
1	358	343	14186	289	35	33	213	2
3	463	238	13462	1013	47	21	200	15
5	547	154	12014	2461	54	14	176	39
10	666	35	5501	8974	67	1	49	166
adapted model	631	70	9843	4632	65	3	158	57
